# Nailfold capillary abnormalities as useful clues in an atypical case suggestive of cancer-associated dermatomyositis sine dermatitis

**DOI:** 10.1093/rap/rkaf043

**Published:** 2025-04-16

**Authors:** Hiroki Kohno, Namiho Irie, Ai Yamane, Tomoko Koura, Kenta Kaneyoshi, Takamichi Sugimoto, Yu Yamazaki, Tomohiro Sugimoto

**Affiliations:** Department of Clinical Immunology and Rheumatology, Hiroshima University Hospital, Hiroshima, Japan; Department of Internal Medicine, Yoshida General Hospital, Akitakata, Japan; Division of Respiratory Medicine and Rheumatology, Faculty of Medicine, Tottori University, Yonago, Japan; Department of Clinical Neuroscience and Therapeutics, Hiroshima University Graduate School of Biomedical and Health Sciences, Hiroshima, Japan; Department of Respiratory Medicine, Miyoshi Central Hospital, Miyoshi, Japan; Department of Respiratory Medicine, Miyoshi Central Hospital, Miyoshi, Japan; Department of Clinical Neuroscience and Therapeutics, Hiroshima University Graduate School of Biomedical and Health Sciences, Hiroshima, Japan; Department of Clinical Neuroscience and Therapeutics, Hiroshima University Graduate School of Biomedical and Health Sciences, Hiroshima, Japan; Department of Clinical Neuroscience and Therapeutics, Hiroshima University Graduate School of Biomedical and Health Sciences, Hiroshima, Japan; Department of Clinical Immunology and Rheumatology, Hiroshima University Hospital, Hiroshima, Japan; Department of Clinical Immunology and Rheumatology, Miyoshi Central Hospital, Miyoshi, Japan

Key message

- Nailfold capillary changes may be useful for the diagnosis of dermatomyositis sine dermatitis.

Dear Editor, We report the case of a 56-year-old woman with chronic dropped head, muscle weakness and dysphagia due to suspected malignancy-associated dermatomyositis. Although typical cutaneous manifestations such as Gottron papules and heliotrope rashes were absent, nailfold capillary abnormalities were integral to the diagnosis of dermatomyositis. This case highlights the importance of nailfold capillaroscopy for dermatomyositis diagnosis, particularly in patients with atypical presentations.

The patient had been diagnosed with rheumatoid arthritis 2 years previously, tested positive for rheumatoid factor and anti-cyclic citrullinated peptide antibodies, and achieved remission with MTX at 12 mg/week. However, the patient was unable to run, raise her extremities, eat, write or button up clothes, in addition to developing dropped head syndrome within 1 year of presenting, resulting in referral to our hospital.

Physical examination revealed a dropped head (Fig. 1A) and muscle weakness in the neck and upper arms. Manual muscle testing showed decreased strength in the neck flexors 2/5, extensors 3/5, deltoids 3/3 and triceps 4/4. Biceps and lower extremity muscle strength were preserved. No muscle tenderness or pathological reflexes were observed. Notably, multiple haemorrhagic spots were observed in the nailfolds (Fig. 1B), and nailfold video-capillaroscopy revealed dilated haemorrhagic capillaries in both hands (Fig. 1C). The patient reported no Raynaud’s phenomenon, and no sclerodactyly, puffy fingers or telangiectasia was observed.

**Figure 1.**
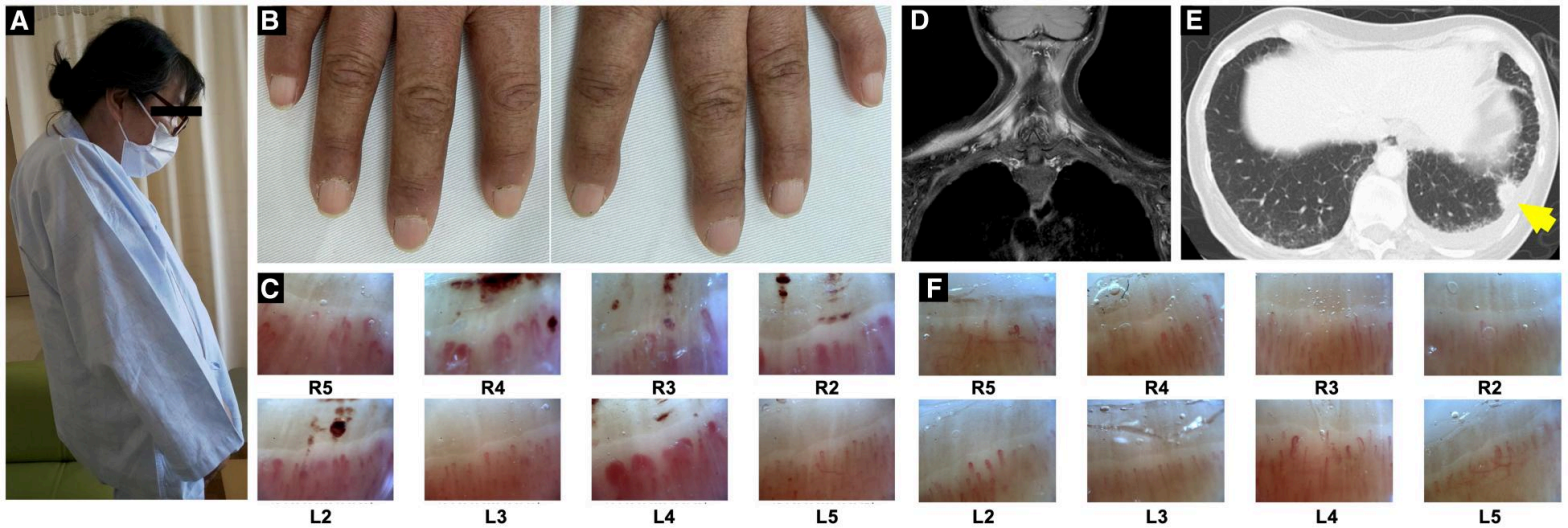
Abnormal findings seen in the patient. (A) Photograph showing the patient’s dropped head. (B) Nailfold capillary abnormalities observed in both hands. (C) Nailfold video-capillaroscopy showing dilated capillaries and haemorrhage (×200 magnification). (D) Coronal magnetic resonance imaging showing contrast enhancement in the trapezius muscle. (E) Computed tomography image showing a nodule (arrow) in the left lower lobe, along with left pleural effusion and interstitial shadowing. (F) Follow-up nailfold video-capillaroscopy performed 4 months after diagnosis showing resolution of haemorrhages and normalization of capillary morphology.

Laboratory tests revealed elevated creatine kinase (389 IU/l), aldolase (8.4 IU/l), lactate dehydrogenase (326 IU/l), C-reactive protein (0.2 mg/dl) and erythrocyte sedimentation rate (48 mm/h). Antinuclear antibody (ANA) was positive at a titre of 1:320, with a nucleolar pattern at 1:320 and a homogeneous pattern at 1:160. Myositis-specific antibodies, including anti-ARS, anti-MDA5, anti-Mi2, anti-TIF1-γ, anti-NXP2 and anti-SAE antibodies, were negative. Tests for anti-centromere, anti-Scl-70, anti-RNA polymerase III, anti-Ku, anti-PM/Scl, anti-ribonucleoprotein, anti-DNA and anti-Sm antibodies were negative.

Magnetic resonance imaging of the neck and shoulders revealed diffuse muscle atrophy and abnormal signals suggestive of myositis (Fig. 1D). Needle electromyography revealed myopathic changes. Chest computed tomography revealed a 20-mm nodule in the left lower lung (Fig. 1E) and pleural effusion. Cell block analysis confirmed the diagnosis of lung adenocarcinoma (Stage IV, with pleural dissemination and metastasis to the left gastric artery trunk lymph node).

The patient was classified as ‘possible’, based on the 2017 EULAR/ACR classification criteria for idiopathic inflammatory myopathies [1]. The patient was thus diagnosed with DM sine dermatitis associated with lung cancer based on nailfold capillary abnormalities, and initiated on glucocorticoid therapy, including intravenous methylprednisolone (1000 mg/day for 3 days), followed by oral prednisolone (50 mg/day, tapered). Chemotherapy with carboplatin, paclitaxel and bevacizumab was initiated for the lung cancer. The myositis responded well to treatment, with normalization of muscle symptoms and CK levels, and improvement in nailfold capillary findings (Fig. 1F).

This case highlights the importance of nailfold capillaroscopy in diagnosing DM, particularly in patients presenting with a dropped head, lacking typical skin manifestations and negativity for myositis-specific antibodies.

Dropped head syndrome is often observed in neurological disorders such as myasthenia gravis, amyotrophic lateral sclerosis and Parkinson’s disease, or as an effect of radiation therapy and aging [2]. In this case, neurological examination, imaging studies and electrophysiological studies did not reveal any findings suggestive of a neurological disorder. In the present case, the nailfold capillary abnormalities and a concomitant malignancy were suggestive of DM. Inflammatory myopathies typically affect the neck flexor muscles more severely than the extensors [3], and dropped head is not frequently reported in PM or DM. Notably, myositis associated with dropped head syndrome often delays diagnosis and is associated with a poor prognosis [4].

Nailfold capillary abnormalities are important clues in diagnosing DM and scleroderma, and reportedly correlate with DM [5, 6]. These abnormalities may be crucial for diagnosis, particularly in cases of atypical dermatitis or myositis presentation that lack skin manifestations. However, further studies are required to investigate their clinical utility.

Recently, DM sine dermatitis has been associated with myositis-specific antibodies [7]. However, implementing a comprehensive myositis-specific antibody clinical test panel remains elusive, making clinical findings, such as nailfold capillary abnormalities, essential for diagnosis.

In this case, malignancy-associated DM was suspected due to the presence of lung cancer lesions. However, all myositis-specific antibodies tested negative. A muscle biopsy was not performed due to severe muscle atrophy, limiting the ability to make a pathological diagnosis. While some diagnostic criteria for DM do not require muscle biopsy, the absence of histological confirmation in this patient necessitates a cautious interpretation of the diagnosis. However, the presence of nailfold capillary abnormalities, which are characteristic microvascular changes associated with DM, may support the diagnosis of DM. The patient’s history of MTX treatment for rheumatoid arthritis might have masked the typical skin manifestations. Nevertheless, this case underscores the importance of nailfold capillaroscopy in diagnosing DM, with subtle or absent skin findings.

## Data Availability

The data underlying this article will be shared on reasonable request to the corresponding author.
